# Endoluminal vacuum-assisted closure (E-Vac) therapy for postoperative esophageal fistula: successful case series and literature review

**DOI:** 10.1186/s12957-020-02073-6

**Published:** 2020-11-14

**Authors:** Carolina Rubicondo, Andrea Lovece, Domenico Pinelli, Amedeo Indriolo, Alessandro Lucianetti, Michele Colledan

**Affiliations:** 1 General Surgery III, ASST Papa Giovanni XXIII, Bergamo, Italy; 2 General Surgery I, ASST Papa Giovanni XXIII, Piazza OMS, 1, 24127 Bergamo, Italy; 3 Department of Gastroenterology and Endoscopy, ASST Papa Giovanni XXIII, Bergamo, Italy

**Keywords:** E-Vac therapy, Oesophageal fistula, Oesophageal leaks, Anastomotic leaks, Bronchogenic cyst

## Abstract

**Background:**

Treatment of esophageal perforations and postoperative anastomotic leaks of the upper gastrointestinal tract remains a challenge. Endoluminal vacuum-assisted closure (E-Vac) therapy has positively contributed, in recent years, to the management of upper gastrointestinal tract perforations by using the same principle of vacuum-assisted closure therapy of external wounds. The aim is to provide continuous wound drainage and to promote tissue granulation, decreasing the needed time to heal with a high rate of leakage closure**.**

**Cases presentation:**

A series of two different cases with clinical and radiological diagnosis of esophageal fistulas, recorded from 2018 to 2019 period at our institution, is presented. The first one is a case of anastomotic leak after esophagectomy for cancer complicated by pleuro-mediastinal abscess, while the second one is a leak of an esophageal suture, few days after resection of a bronchogenic cyst perforated into the esophageal lumen. Both cases were successfully treated with E-Vac therapy.

**Conclusion:**

Our experience shows the usefulness of E-Vac therapy in the management of anastomotic and non-anastomotic esophageal fistulas. Further research is needed to better define its indications, to compare it to traditional treatments and to evaluate its long-term efficacy.

## Background

Esophageal perforations and postoperative esophageal anastomotic leaks are still a life-threatening condition; the reported mortality ranges from 10 to 25%, when therapy is started within 24 h, and from 40 to 60%, when the treatment is delayed [1]. Iatrogenic perforation is the leading cause of esophageal perforations, accounting for around 60% of all cases. Less frequent causes are trauma at the upper abdomen or chest, Boerhaave’s syndrome, or spontaneous perforations induced by straining and vomiting [2]. Esophageal anastomotic leak remains one of the most devastating complications after esophagectomy and gastrectomy, with a wide range of reported incidences from 0 to 35% after esophagectomy and 2.7 to 12.3% after total gastrectomy [3]. The key point of a correct treatment includes resuscitation of the patient, assessment of the defect, and timely decision-making [4, 5]. Surgical revision is usually challenging and carries a high risk of severe secondary complications. In the last few years, endoscopy has gained a primary role in both diagnosis and treatment of esophageal perforations and leaks. Several minimally invasive treatments have become available, including application of metal clips, fibrin glue, and placement of self-expanding metal or plastic stents [6]. However, these treatments may not always lead to a sufficient sealing of the leakage.

A promising method to manage anastomotic leaks and perforations is the endoscopic vacuum-assisted closure system. Vacuum-assisted therapy is broadly used in the management of skin and muscular defects, the first report dating back to 1962 [7]. The permanent suction reduces wound secretion and edema, improves microcirculation, induces granulation of the wound, and decreases wound size by retraction. Since the 1990s, the number of indications for VAC therapy has been steadily increasing [8, 9], more recently including its endoscopic application to perforations and fistulae of the digestive tract, mainly in the rectum and esophagus.

This paper reports the successful closure of two different kinds of esophageal fistula; the first one is a complex anastomotic leak, complicated by pleuro-mediastinal abscess, after esophagectomy for cancer, which is the most common indication for this treatment. The second one is a leak of esophageal suture, few days after resection of a rare bronchogenic cyst perforated into the esophageal lumen.

### The E-Vac method

**Fig. 1:**
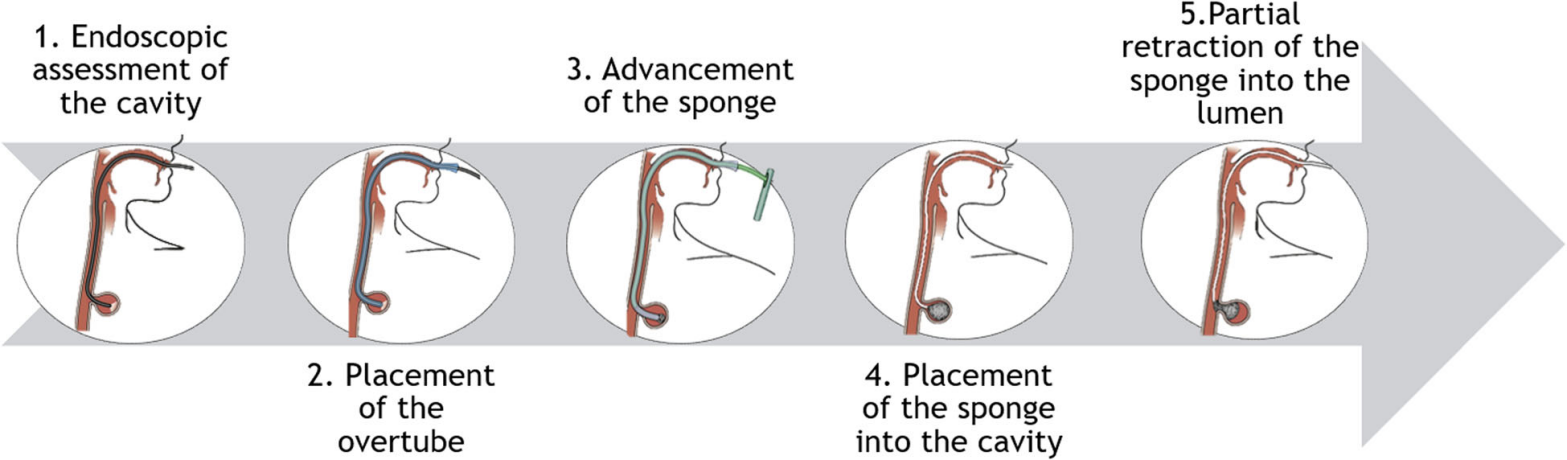
Pathway of correct Eso-Sponge® placement

The E-VAC^9^ system for different types of postoperative esophageal leak consists in a sponge, to be placed across the defect, connected by a tube to an external vacuum pomp and a reservoir (ESO-Sponge®). The application system includes an application tube and a pusher. The treatment is performed in the operating theater with the patient in a supine position, anesthetized and intubated. General anesthesia is required to facilitate insertion of the E-Vac device. First, the fistula cavity is explored and measured with a flexible endoscope. The application tube (overtube) is pushed over the endoscope and brought up to the distal end of the leakage cavity. The endoscope is retracted, and the sponge is pushed into the overtube to its end. A metallic marker placed on the skin may help in the positioning and avoid sponge displacement. The application system is then removed, and the sponge tubing is passed and exited through the nostril and connected to the vacuum pump. Continuous suction with a negative pressure of 125 mm Hg is applied. Figure 1 provides a pathway for a correct placement of the sponge. In accordance with the experience of VAC therapy for skin defects, the procedure is repeated by replacing the sponge every 72 h, until granulation leads to obliteration of the cavity.

## Case presentation 1

A 62-year-old woman diagnosed with esophageal cancer was referred to our hospital. Her work up included esophagogastroduodenoscopy (EGDS), contrast-enhanced computed tomography (CT) scan and ^18^F-fluorodeoxyglucose-positron emission tomography (^18^F-FDG-PET), leading to the diagnosis of esophageal adenocarcinoma (cT3N+M0) localized from 34 to 37 cm from the incisors, with the EGJ at 39 cm (Siewert 1). After a multidisciplinary discussion, she received two cycles of neoadjuvant chemotherapy, consisting in docetaxel, cisplatin, and 5-fluorouracil, in 3-week intervals. Post-treatment CT scan revealed a partial response according to RECIST criteria, with a reduction in both primary tumor size and lymphadenopathies (post-treatment staging: cT2N+). The patient was then scheduled for an Ivor-Lewis esophagectomy, with peri-gastric and peri-esophageal lymphadenectomy and an intrathoracic, end-to-end, semimechanical anastomosis.

**Fig. 2:**
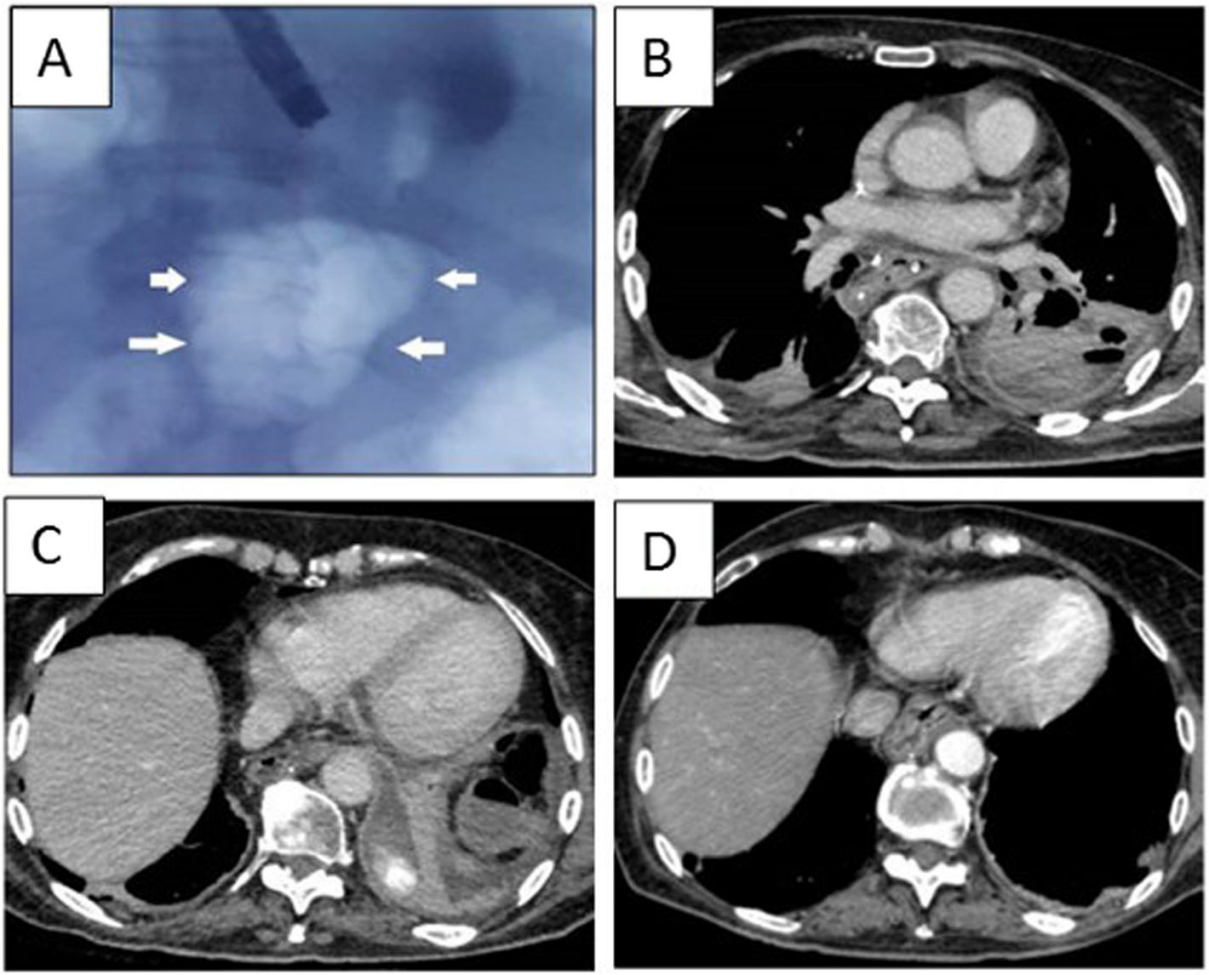
**a** Radiologic view of the paraesophageal abscess (white arrows) during endoscopic examiation. **b**, **c** CT scan showing a left pleuro-mediastinal abscess. **d** A 3-month follow-up.

Post-operative course was normal until postoperative day 9, when she developed fever and white cell count rose to 21 k/mm^3^. Chest CT scan revealed a frank anastomotic leak with a periesophageal, loculated abscess formation with a maximum thickness of 14 mm × 78 mm (Fig. 2). Broad-spectrum, empiric antibiotic coverage was initiated in the form of Piperacillin-Tazobactam every 8 h. EGDS confirmed a > 1-cm disruption at the gastroesophageal anastomosis, without a gastric conduit necrosis.

**Fig. 3:**
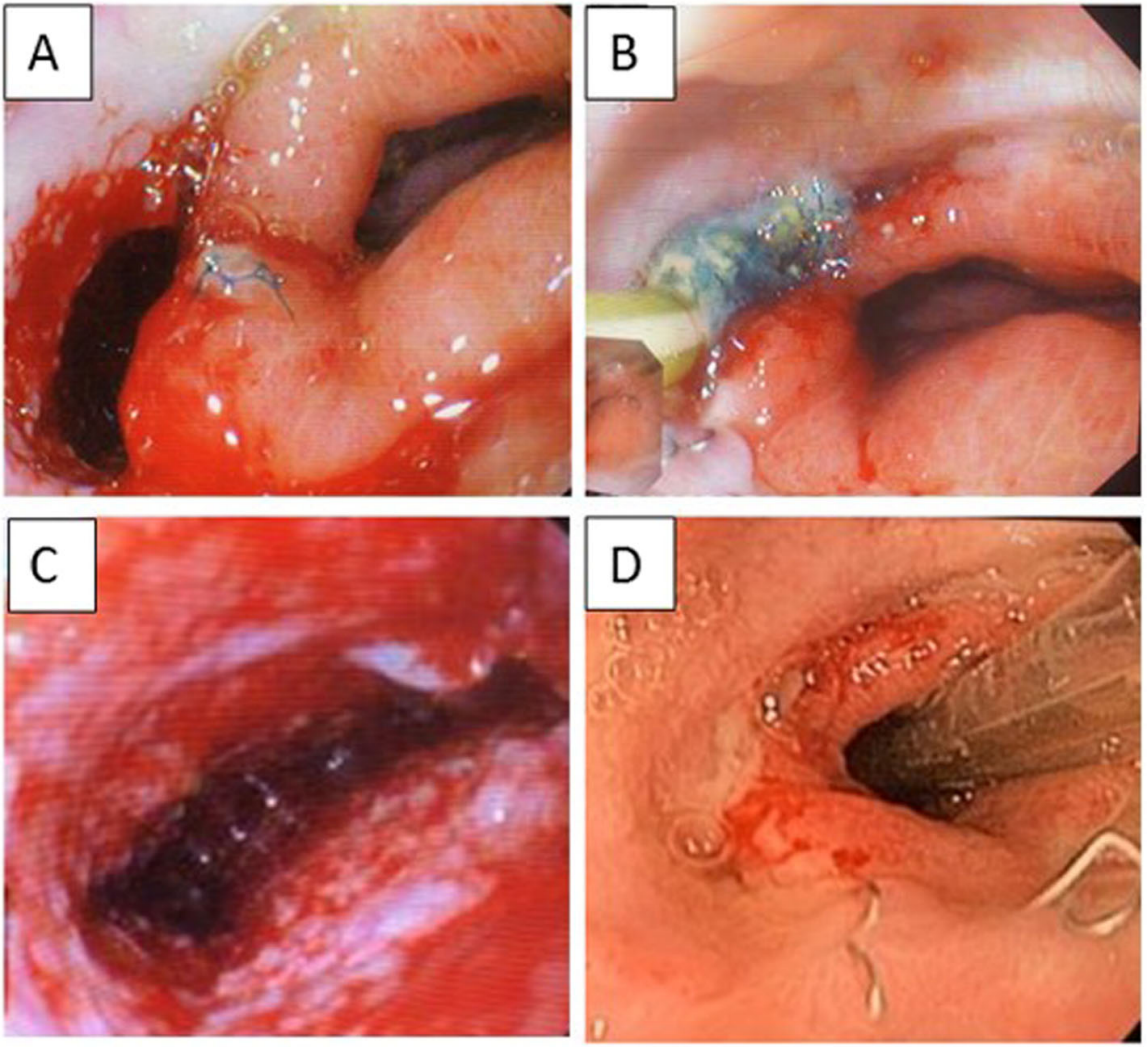
**a** Anastomotic leak. **b** Eso-sponge® placement into the fistula. **c** Granulation tissue during the treatment. **g** A 3-month follow-up (nasogastric tube in place)

Usually, in our experience, standard endoscopic treatment consisted of external drainage, endoscopic lavage, debridement of the fistula, and, if possible, implantation of covered stents. Unfortunately, in this case, the pleuro-mediastinal abscess was very difficult to reach by external drainages and the anastomotic leakage was very high. With the aim of resolving the leakage and controlling the abscess, we opted for E-Vac treatment. At the beginning of our experience, we used to treat with E-Vac therapy only to perforations that had a diameter > 1 cm, placing the sponge into the cavity and then shaping it according to the size of the defect, measured with the standard endoscope (diameter of 9 mm) or the pediatric one (diameter of 5 mm). Now, we use E-Vac therapy also for defects smaller than 1 cm, shaping the sponge accordingly or placing it directly into the esophageal lumen. For the present case, E-Vac therapy was performed for a total of 22 days, while the sponge was changed 6 times. During the first three changes, the sponge was placed directly into the cavity and then shaped, retracted, and switched to intraluminal treatment. Excellent granulation of the tissues around the leak was achieved, together with a progressive reduction of the cavity size and of the pleuro-mediastinal abscess (Fig. 3). Daily output from the collection bag dropped from 200 ml during the first 2 changes to less than 30 during the last 2. We usually perform a jejunostomy during every Ivor-Lewis esophagectomy, so patient’s nutrition was guaranteed during the whole period. Patient conditions dramatically improved with a rapid drop of the fistula output, a normalization of temperature and white cell count, and no sign of sepsis. At the end, both an endoscopic and radiologic check confirmed the closure of the leak. The recovery was then uneventful and oral nutrition was re-administered. The patient was discharged without sign of recurrence 3 months after the initial surgery. A follow-up endoscopy, performed 1 year after surgery, showed no sign of recurrence or esophageal stricture.

## Case presentation 2

**Fig. 4:**
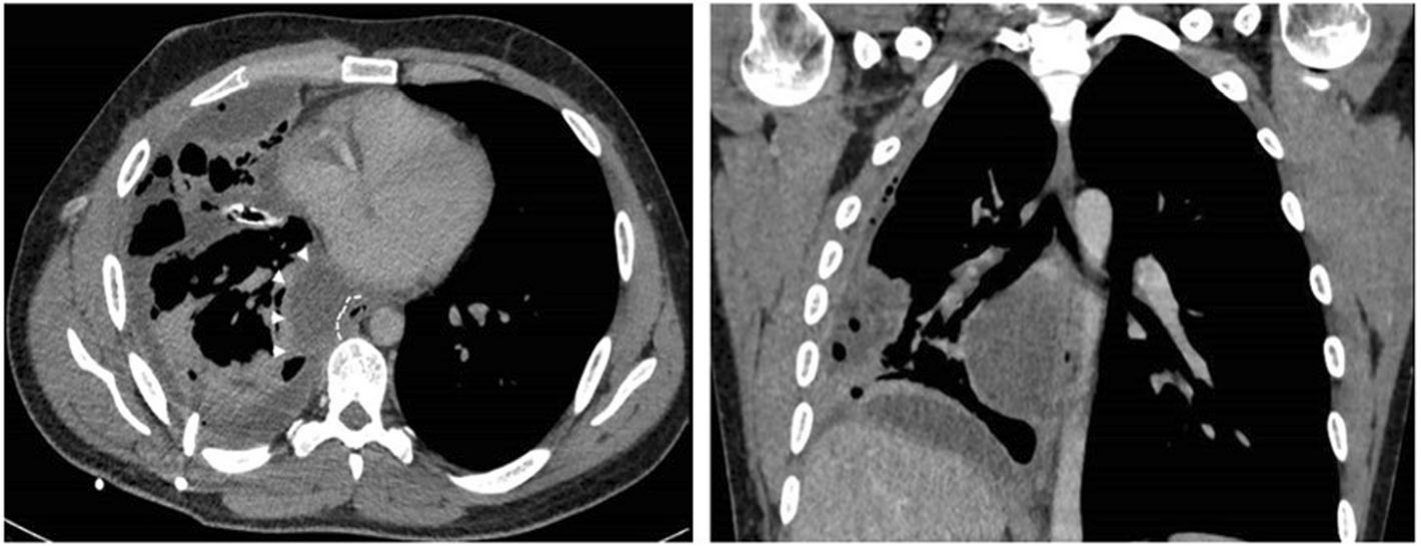
CT scan with a massive pleural effusion in the right hemithorax and complete atelectasis of the ipsilateral lung

A 25-year-old man was referred to our hospital in 2014 for a suspected right bronchogenic cyst. He underwent a trans-esophageal biopsy to confirm the diagnosis. After a few days, he started to complain of fever and acute chest pain. A contrast-enhanced CT scan revealed a massive pleural effusion in the right hemithorax with complete atelectasis of the ipsilateral lung (Fig. 4).

A right thoracoscopy was performed, in order to achieve a complete debridement and drainage. During the procedure, no esophageal perforation was seen. The following clinical course was normal, so the patient was discharged on postoperative day 15.

**Fig. 5:**
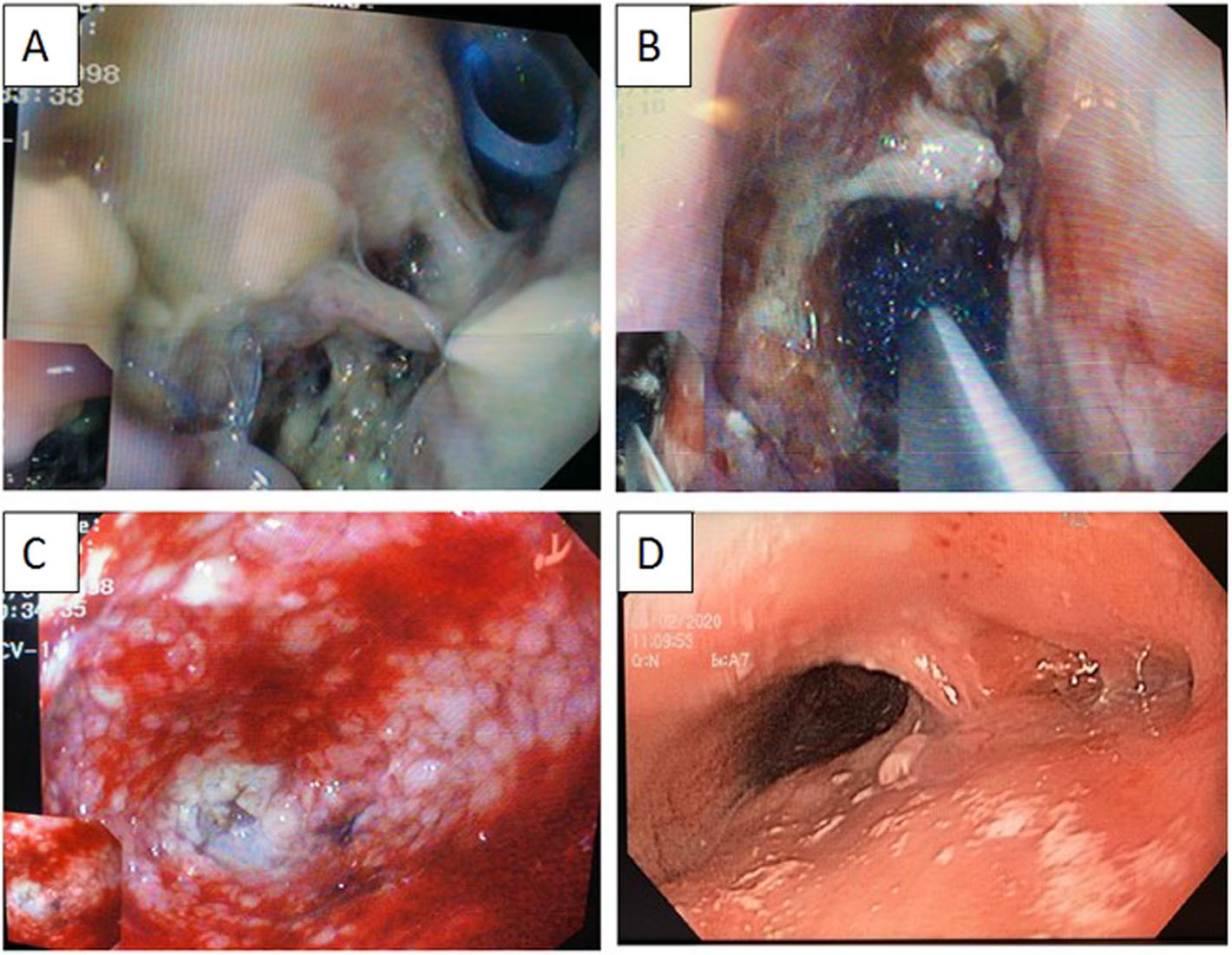
**a** Post-surgical leak with the thoracic drain in place. **b** Positioning of the Eso-sponge® into the cavity. **c** Granulation tissue during the treatment. **d** A 3-month follow-up

In December 2019, the patient was referred to our Emergency Department because of the sudden onset of fever (39 °C) with acute chest pain. He underwent a CT-scan which revealed a suspected esophageal perforation with acute mediastinitis and right pleural effusion. A subsequent gastroscopy showed, at 35 cm from dental arch, an erosion of the esophageal wall 1,5-cm long and a 2-mm perforation with a small leak of purulent liquid. Furthermore, an endoscopic ultrasound revealed the presence of the bronchogenic cyst just outside the erosive area. Thus, a right thoracotomy with intraoperative endoscopy was performed. An esophageal perforation at the level of the cyst was found, so the right hemithorax was cleaned, the cyst was opened in order to better understand its margin, and then resected, while the esophageal wall was closed with two interrupted, absorbable stitches. A 24-Ch drain was left in place. After 2 days, salivary material appeared into the drain, so the patient underwent an EGDS, which revealed a 7-mm hole of the esophageal wall at the level of the previous suture (Fig. 5a). Thus, an E-Vac therapy was placed directly into the perforation (Fig. 5b) only during the first placement, with the aim of cleaning the mediastinum and healing the esophageal wall, which was locally compromised by the abscess. Patient’s nutrition was guaranteed by a nasojejunal feeding tube.

The duration of the endoscopic treatment was 17 days, the sponge was changed 5 times (Fig. 5c, d). After the removal of the last sponge, the patient started an oral diet without complications. One last barium swallow study showed no leak, so the patient was discharged home. Pathology confirmed the diagnosis of bronchogenic cyst. At a 3-month follow-up, there was no sign of recurrence.

## Discussion and literature review

The present paper describes two different cases of esophageal fistulas, with different origin and frequency, successfully managed by E-Vac therapy.

Regarding case no. 1, postoperative esophageal anastomotic leak is a severe condition that negatively impacts on postoperative outcomes [10]. The incidence ranges between 0 and 35% [3] after esophagectomy and 2.7 to 12.3% after total gastrectomy with up to 60% of mortality [11].

As regards case no. 2, only few bronchogenic cysts communicating with the esophagus have been reported in the available literature [12]. Only one case of bronchogenic cyst complicated with post-operative esophageal perforation was reported [13].

The management of esophageal fistula, regardless the cause, requires a multidisciplinary approach between surgeons, gastroenterologists, radiologists, and intensive care physicians. The key point of treatment is the control of sepsis, achieved by containing the ongoing leakage from the esophagus, draining the pleural, mediastinal or abdominal cavities, and giving appropriate antibiotic therapy. Nutritional support is mandatory, preferably with the enteral way or via parenteral nutritional when the former is not available [14]. Surgical closure of the esophageal defect is generally complex and scarcely effective. On the other hand, during the last decades, numerous endoscopic techniques have been developed, the real cornerstone of which being the endoscopic stent implantation [15], with a demonstrated clinical success rate of over 80%. In 2008, Weidenhagen et al. [16] reported the first Endo-VAC treatment of anastomotic leakages after rectal resections. Only a few years later, Loske et al. started to transfer this treatment in patients with leakages in the upper gastrointestinal tract [17].

In the last decade, the E-Vac therapy for the treatment of upper GI defects has become a valid endoscopic alternative. This has been demonstrated in published case series of more than 200 patients, in numerous German endoscopic centers [18].

In 2017, Kuehn et al. [19] published a MEDLINE analysis of 11 case series with over 210 patients with upper GI tract defects treated with E-Vac. In this review, success rate was 90 and 96% for anastomotic leakages and esophageal perforations, respectively. Currently, there are no prospective randomized clinical trials available comparing endoscopic stenting, E-Vac therapy, and surgical revisions in upper GI leakages or perforations. Another crucial point is that there are no current guidelines regarding the indication for E-Vac therapy, the minimum diameter of treatable perforations, or a standard treatment duration. So far, data are restricted to single-center, non-randomized trials; the biggest monocentric studies were published by Laukoetter et al. [20] and Bludau et al. [21]. They divided the defects by size: small (< 10% of circumferential involvement), intermediate (10–40%), and large (> 45%). However, they did not analyze the possible correlations between the size of the defect and the indication and duration of therapy.

Our initial practice in E-Vac therapy for treatment of esophageal postoperative fistula confirms some technical rules:

- Crucial for E-Vac placement is the dimension of the esophageal defect: it should be crossed with a standard diagnostic endoscope or a pediatric one in order to understand its dimensions.
- Granulation tissue often ingrowths into the sponge, leading to more difficult removal. More frequent changes of the device reduce the risk of bleeding and the complexity of the sponge extraction. We always changed the sponge within 72 h without problems.
- Enteral feeding should be the mainstay of nutrition support (by nasojejunal feeding tube, PEG, or surgical jejunostomy); parenteral nutrition should be established as a bridge strategy.

## Conclusion

Our cases confirm that the open-cell sponge together with the topical application of negative pressure helps sealing the leak while providing an additional and simultaneous drainage of the cavity distal to it. This transluminal drainage allows an effective and continuous drainage of the abscess cavity, which is often difficult to address radiologically, therefore controlling and reducing the sepsis.

Both our cases, despite different pathologies and type of perforations, were successfully treated with the E-VAC therapy, confirming its promising prospects for all kind of esophageal perforations, combined with a correct treatment of sepsis and risk of malnutrition. Further analysis with a larger number of patients should be performed to determine the correct indications for E-Vac therapy.

## Data Availability

All data generated or analyzed during this study are included in this published article

## Abbreviations

VAC: Vacuum-assisted closure
EGDS: Esophagogastroduodenoscopy
CT: Computed tomography
PET: Positron-emission tomography
